# Auditory Statistical Learning Deficits in Children with Autism Spectrum Disorder: A Comparison of Visual and Auditory Segmentation Paradigms

**DOI:** 10.3390/jintelligence14090195

**Published:** 2026-09-01

**Authors:** Yunhuan Pu, Wei Zhao

**Affiliations:** 1 Faculty of Education, Shaanxi Normal University, Xi’an 710062, China; pyh1022@163.com; 2 College of Teacher Education, Sichuan University of Arts and Science, Dazhou 635000, China

**Keywords:** children with autism, visual statistical learning, auditory statistical learning

## Abstract

This study involved 30 children with Autism Spectrum Disorder (ASD) aged 7–10 years and 31 typically developing (TD) children who were matched for age and IQ. The participants’ performance on visual and auditory statistical learning tasks within a segmentation paradigm was assessed. Generalised linear mixed models (GLMMs) and linear mixed models (LMMs) were used to analyse response accuracy and reaction times, respectively. The aim was to explore the characteristics of statistical learning in children with ASD. The results showed that the children with ASD failed to perform above chance level on the auditory statistical learning task (*p* = 0.156). Only 40% of ASD participants performed better than expected by chance. Their performance was significantly lower than that of the TD children. Conversely, children with ASD performed significantly above chance level in the visual statistical learning task (*p* < 0.001). GLMM analysis revealed a significant interaction between group and task modality (*p* = 0.023). Specifically, children with ASD demonstrated significantly lower accuracy in auditory statistical learning than TD children (*p* < 0.001). In contrast, no significant difference was observed between groups in the visual task (*p* = 0.468). LMM analysis showed a significant interaction between group and task modality regarding reaction times (*p* = 0.020). Children with ASD exhibited significantly longer reaction times than TD children in both the visual and auditory tasks (*ps* < 0.001). These findings suggest that children with ASD have difficulty with auditory statistical learning, but perform relatively well in visual tasks within the segmentation paradigm.

## 1. Introduction

Statistical learning (SL) refers to the human ability to unconsciously detect and leverage patterns in the environment, representing a form of implicit learning (Saffran et al., 1999). Specifically, it involves the brain’s automatic extraction of probabilistic regularities and patterns from continuous streams of visual, auditory, and tactile information without intentional rule-seeking. This mechanism plays a pivotal role in the acquisition of word segmentation (Saffran et al., 1996), semantic knowledge (Romberg & Saffran, 2010), and early literacy skills (Qi et al., 2019).

Autism Spectrum Disorder (ASD), also referred to as autism, is a neurodevelopmental disorder characterized by deficits in social communication and interaction, alongside restricted, repetitive patterns of behavior and interests. Research indicates a positive correlation between visual statistical learning (VSL) performance in children with ASD and their language and social abilities. In contrast, auditory statistical learning (ASL) performance is negatively correlated with the core symptoms of autism, meaning that more severe symptoms are associated with lower ASL scores (Parks et al., 2020). The study demonstrated that neural activity associated with ASL in infancy, especially in the left temporal lobe, significantly predicts expressive language ability and autism symptom severity at 36 months, suggesting that ASL plays a specialized role in the language development of individuals with ASD (Liu et al., 2021). However, current research on the SL ability of individuals with ASD remains controversial. Some studies suggest that SL is preserved in children with ASD (Barnes et al., 2008; Brown et al., 2010), while others report significant differences compared to typically developing (TD) children (Jeste et al., 2015; Scott-Van Zeeland et al., 2010). Additionally, some findings indicate that children with ASD generally exhibit longer reaction times in completing SL tasks than their TD peers (Treves et al., 2023; Parsons & Baron-Cohen, 2023). These discrepancies may be attributed to the influence of audiovisual modalities, as some studies have focused exclusively on either the visual or auditory channel. Furthermore, such variations may stem from differences in participants’ cognitive and linguistic profiles (Parks et al., 2020; Yim & Yang, 2021) or from inconsistencies in the experimental paradigms employed.

Common paradigms for measuring SL include the segmentation paradigm, artificial grammar learning (AGL), the serial reaction time (SRT) task, the Hebb learning paradigm, the contextual cueing task, and various cue-reaction time tasks. Among these, the segmentation paradigm (also known as the triplet task) has been shown to effectively simulate the word segmentation process. With appropriate adjustments, it can be applied to studies involving diverse age groups and populations, making it one of the mainstream paradigms widely utilized in the field of SL in recent years (Kligler & Gabay, 2023). Therefore, this study recruits children with ASD (aged 7–10 years) who have an IQ above 70 and demonstrate no significant differences in language ability compared to TD children. The present study aims to investigate the characteristics of SL in children diagnosed with ASD by employing visual and auditory SL tasks based on the segmentation paradigm. This study proposed four hypotheses: (a) lower auditory statistical learning in ASD, (b) preserved visual statistical learning, (c) generally slower reaction times in ASD, and (d) a group-by-modality interaction.

## 2. Method

### 2.1. Participants

Participants with ASD were recruited through community advertising and referrals from local autism rehabilitation centers. Inclusion criteria were: (a) a formal diagnosis of autism confirmed by a qualified clinician in accordance with DSM-5 criteria; (b) a score between 30 and 36.5 on the Childhood Autism Rating Scale (CARS), indicating mild to moderate symptom severity; (c) a score of 70 or above on the Combined Raven’s Test (CRT); (d) age between 7 and 10 years; and (e) no uncorrected visual or auditory impairments.

A total of 30 children with ASD (24 males, 6 females) met these criteria. All participants were enrolled in Grades 1–3 within mainstream schools and came from families where Mandarin was the primary language spoken. Assessments were conducted in Mandarin.

Diagnoses were further confirmed using the Autism Diagnostic Observation Schedule—Second Edition (ADOS-2) and/or the Autism Diagnostic Interview—Revised (ADI-R), administered by trained research clinicians. None of the participants had a history of psychotropic medication use. Participants did not exhibit any comorbidities, including but not limited to learning disabilities, ADHD, emotional disorders, or epilepsy. None of the participants had prior exposure to similar statistical learning tasks.

TD children were recruited based on the criteria: (a) a score greater than 70 on the CRT; (b) ages ranging from 7 to 10 years; and (c) no visual or auditory impairments. The control group comprised 31 TD children (20 boys and 11 girls). A chi-squared test revealed no significant difference in gender distribution between the two groups (*χ*^2^(1) = 1.818, *p* = 0.178), thus ensuring the comparability of the groups.

Receptive vocabulary abilities were assessed using the Peabody Picture Vocabulary Test-Revised (PPVT-R). A group-matching strategy was employed, and the quality of matching was quantified using the standardized mean difference (SMD; Cohen’s *d*) with 95% confidence intervals (CIs). Group comparisons indicated no significant differences in age, IQ, or PPVT-R scores between the groups (all *ps* > 0.05). The absolute SMD values were all below 0.5, and their 95% CIs included zero, indicating that the matching was satisfactory (see Table 1).

### 2.2. Experimental Materials

The experimental design was adapted from van Witteloostuijn et al. (2019) and Kidd et al. (2020), using 12 alien images and 12 nonsense syllables as stimuli. A pilot study was conducted with five TD children and three children with ASD (aged 7–10). During the pilot, participants often became distracted (e.g., looking around) toward the end of the familiarization phase. Additionally, a notable increase in random, rapid responses emerged toward the end of the test phase, particularly among children with ASD. To reduce cognitive load and test duration, the task was modified. Rather than simply reducing the duration, which could impair perception during the familiarization phase, the number of stimuli was reduced. As stimuli were organized into triplets, removing only one or two stimuli would disrupt the consistency of transitional probabilities between groups. Therefore, one full triplet (3 stimuli) was removed from both tasks. To maintain equivalence between the two modalities, an identical number of stimuli were removed, and the ratio of target to distractor materials was kept consistent. Before the adjustment, each task comprised 12 stimuli (images or audio), forming 4 target triplets and 4 distractor triplets. After the adjustment, both tasks were streamlined to 9 stimuli, forming 3 target triplets and 3 distractor triplets. This change reduced the test duration while maintaining the transition probabilities. Participants in the pilot study (*n* = 8) were excluded from the main analysis.

Both tasks were programmed using E-Prime 3.0. In the VSL task, 9 colored images (labeled A through I) were organized into 6 potential triplets: target triplets (ABC, DEF, GHI) and distractor triplets (BFG, HAD, FIC). For the ASL task, 9 nonsense syllables (ti, ma, bo, di, pa, he, ki, lo, ga) were arranged into triplets, with target triplets (pa–ti–he, bo–ma–ki, ga–di–lo) and distractor triplets (lo–ki–pa, ti–ga–ma, he–bo–di). All syllables used in the ASL materials were in the first tone (Tone 1). They were recorded by a female native Mandarin speaker with a Level 2, Grade A certification in the National Mandarin Proficiency Test, in a soundproof recording booth with background noise below 30 dB. The recordings were edited and concatenated into three-syllable sequences using Audacity (version 3.4.2, a free open-source audio editing software).

### 2.3. Experimental Procedure

The experiment was conducted individually in a quiet classroom or at participants’ homes. Stimuli were presented on a 14-inch laptop screen with a resolution of 2160 × 1440. Before the experiment, task instructions were explained to participants, and responses were collected via an external keypad featuring only the “F,” “J,” and “Space” keys. The experiment consisted of a familiarization phase followed by a test phase. The familiarization phase used an 800-ms inter-triplet interval, rendering the task a segmented paradigm with explicit intervals, rather than the continuous streams typical of classical statistical learning studies. In this study, “trial” is defined differently across phases. A familiarization trial involves presenting one triplet, while a test trial consists of a forced-choice comparison between a target and a distractor triplet. For the visual tasks, the viewing distance was set to 50–70 cm, and the screen brightness was set to 100 cd/m^2^. For the auditory tasks, all participants used identical headphones.

### 2.4. Visual Statistical Learning (VSL) Procedure

**Figure 1:**
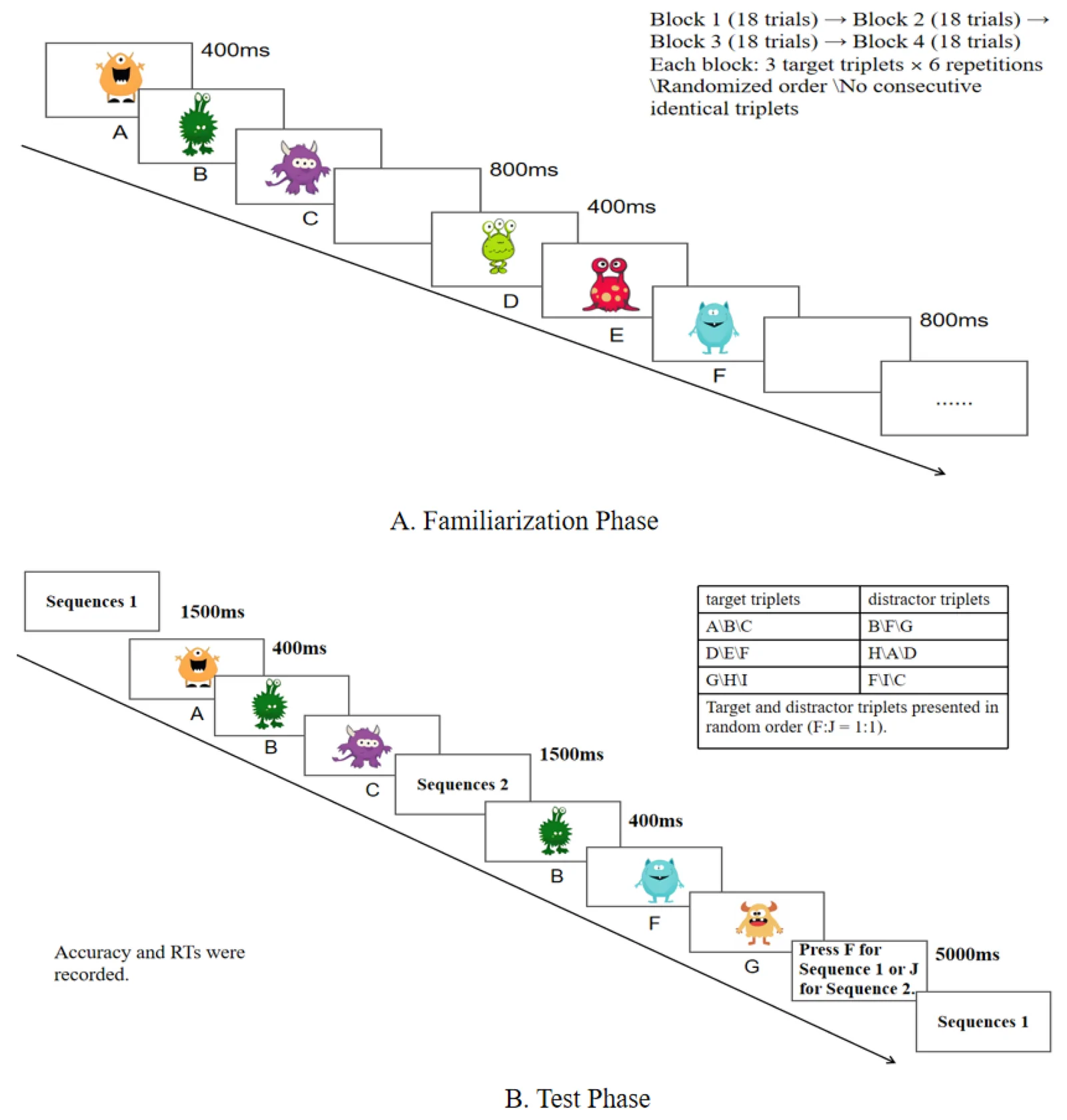
Schematic representation of the familiarization phase and the test phase in the Visual Statistical Learning (VSL) task.

During the familiarization phase, nine stimulus images (A–I) were presented sequentially. Each image appeared for 400 ms, with each triplet lasting 1200 ms, followed by an 800 ms inter-stimulus interval (ISI). The three target triplets (ABC, DEF, GHI) were each repeated 24 times, totaling 72 trials (~3 min). To reduce participant fatigue, these trials were divided into four blocks of 18 trials each, corresponding to the three target triplets repeated six times, with a 30-s rest period between blocks. Within each block, the order of triplets was fully randomized, with the constraint that no identical target triplet appeared consecutively. Figure 1A illustrates the stimulus presentation sequence during the VSL familiarization phase.

During the test phase, the same nine images from the familiarization phase were used to construct three distractor triplets: BFG, HAD, and FIC. Presentation parameters for the distractor triplets matched those of the target triplets (400 ms per image; 1200 ms per triplet). Target and distractor triplets were presented in a randomized order. Each trial began with the appeared of the labels “Sequence 1” and “Sequence 2” for 1500 ms, followed by a prompt asking participants to identify the more familiar sequence (e.g., press ‘F’ for Sequence 1 or ‘J’ for Sequence 2). The prompt remained on screen for 5000 ms, advancing automatically if no response was made. The test consisted of 27 trials (9 target–distractor combinations × 3 repetitions), with accuracy and reaction times (RTs) recorded (Figure 1B). Before the test, participants completed five practice trials with on-screen feedback and were required to respond correctly to at least three of these to proceed.

### 2.5. Auditory Statistical Learning (ASL) Procedure

**Figure 2:**
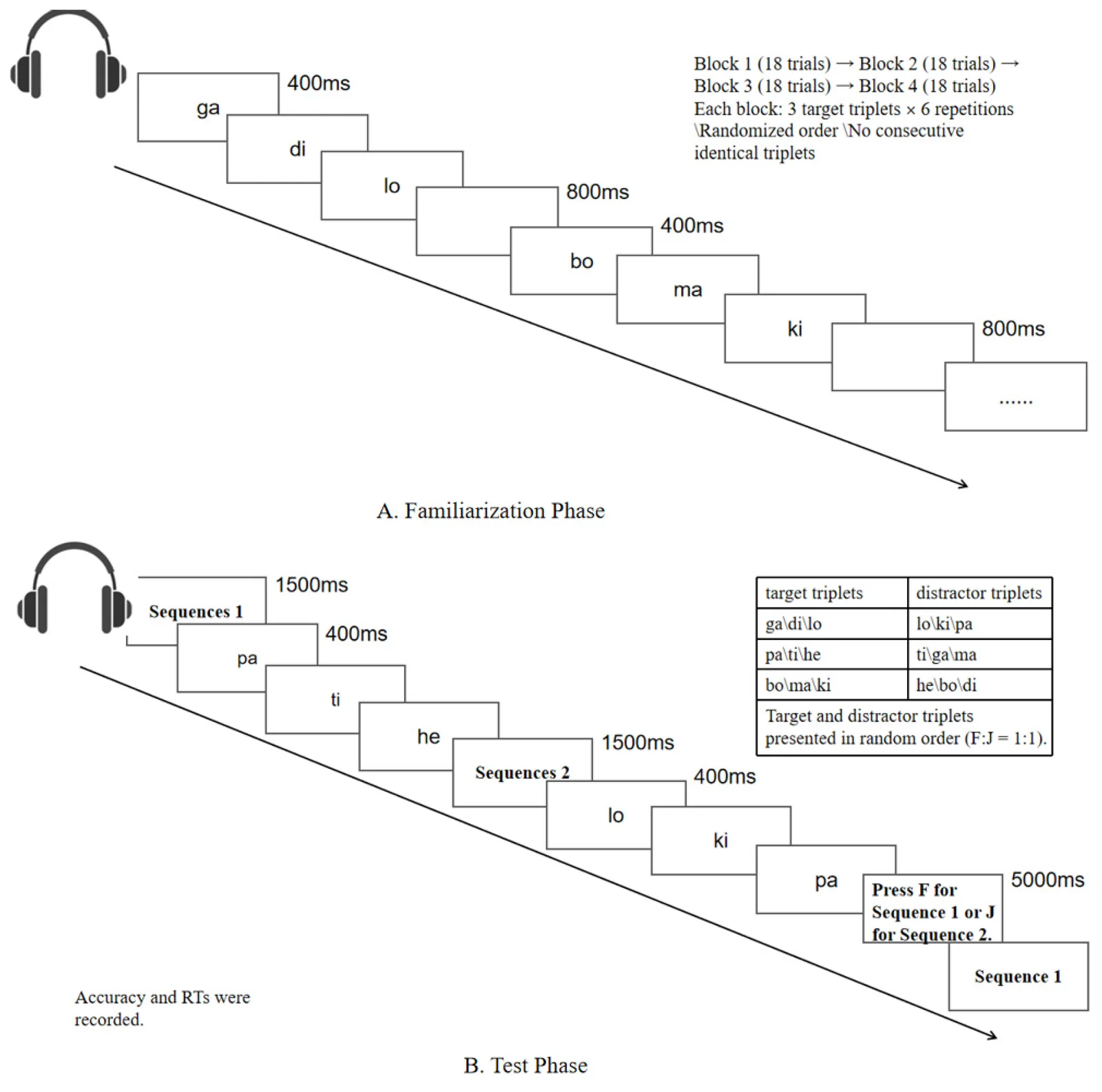
Schematic representation of the familiarization phase and the test phase in the Auditory Statistical Learning (ASL) task.

During the familiarization phase, nine syllables (“ti,” “ma,” “bo,” “di,” “pa,” “he,” “ki,” “lo,” “ga”) were presented auditorily. Each syllable lasted 400 ms, with each triplet lasting 1200 ms, followed by an 800 ms inter-stimulus interval (ISI). The three target triplets (“pa-ti-he,” “bo-ma-ki,” “ga-di-lo”) were each repeated 24 times, totaling 72 trials (~3 min). To reduce fatigue, these trials were divided into four blocks of 18 trials each (corresponding to the three target triplets repeated six times), with a 30-s rest period between blocks. Within each block, triplet order was fully randomized, with the constraint that no identical target triplet appeared consecutively. Figure 2A illustrates the stimulus presentation sequence during the ASL familiarization phase.

During the test phase, the same nine syllables from the familiarization phase were used to construct three distractor triplets: “lo-ki-pa,” “ti-ga-ma,” and “he-bo-di.” Presentation parameters for the distractor triplets matched those of the target triplets (400 ms per syllable; 1200 ms per triplet). Target and distractor triplets were presented in randomized order. Each trial began with the labels “Sequence 1” and “Sequence 2” for 1500 ms, followed by a prompt asking participants to identify the more familiar sequence (e.g., press ‘F’ for Sequence 1 or ‘J’ for Sequence 2). The prompt remained on screen for 5000 ms, advancing automatically if no response was made. The test comprised 27 trials (9 target-distractor combinations × 3 repetitions), with accuracy and RTs recorded (Figure 2B). Before the test, participants completed five practice trials with on-screen feedback and were required to respond correctly to at least three of these to proceed.

## 3. Experimental Design

The current study employed a 2 (Group: ASD vs. TD) × 2 (Modality: VSL vs. ASL) mixed design, with Group as a between-subjects factor and Modality as a within-subjects factor. Dependent variables were accuracy and RTs. To control for order effects, task sequence and stimulus presentation were counterbalanced. Odd-numbered participants completed the visual task first before the auditory task, and even-numbered participants completed the auditory task first, with a brief rest between tasks. The test phase consisted of 27 trials (9 target–distractor combinations × 3 repetitions), presented in a fixed pseudorandom sequence with target and distractor positions evenly distributed. Task order was included as a covariate, and triplet type was modeled as a random intercept to account for triplet-specific variance.

## 4. Data Analysis and Processing

Data were analyzed using R (version 4.5.3) with the *lme4*, *lmerTest*, and *emmeans* packages. Mixed-effects models were employed in place of traditional repeated-measures ANOVA, as they do not require sphericity, handle missing data and unbalanced designs more effectively, and provide more robust estimates (Zhou et al., 2024). Categorical predictors were dummy-coded, with the TD children and the VSL task as the respective reference levels for Group and Modality.

### 4.1. Analysis of Accuracy

Response accuracy was analyzed using a generalized linear mixed model (GLMM) with a binomial distribution and a logit link function. Fixed effects included group, modality, and their interaction, with age, IQ, and PPVT-R scores as covariates. Regarding the random effects structure, a comparison was conducted between a model with random intercepts for participants and one that also incorporated random slopes for modality. Likelihood ratio tests indicated that the random-slope model provided a significantly better fit (χ^2^(2) = 14.96, *p* < 0.001) and yielded a lower AIC (4282.17) compared to the random-intercept model (4293.13). The final model included random slopes and was estimated using maximum likelihood (ML) with a maximum of 200,000 iterations. To decompose significant interaction effects, simple effects analyses were conducted using the *emmeans* package, with Tukey’s HSD correction applied for multiple comparisons.

Model formula: logit(*pijk*) = *β*0 + *β*1 ⋅ Group*i* + *β*2 ⋅ modality*j* + *β*3 ⋅ (Group × modality)*ij* + *β*4 ⋅IQ*i* + *β*5 ⋅age*i* + *β*6 ⋅PPVT*i* + *u*0*i* + *u*1*i* ⋅modality*j*

In this formulation, *pijk* denotes the probability of a correct response for the *i*-th participant in the *k*-th trial of the *j*-th modality. The terms *u*0*i* and *u*1*i* represent the random intercept for participants and the random slope for task modality, respectively. All other terms are as defined in the preceding model equation.

### 4.2. Analysis of Reaction Time

RTs were positively skewed and log-transformed (logRT) before analysis. RTs from correct trials were trimmed by excluding responses shorter than 200 ms and outliers beyond ±3 SD from the mean, representing 3.86% of responses in children with ASD and 2.07% in TD children. Linear mixed-effects models (LMMs) were fitted using restricted maximum likelihood estimation (REML) with Satterthwaite approximation for degrees of freedom. Fixed effects included group, modality, interaction, and the covariates (age, IQ, and PPVT-R), consistent with the GLMMs. Regarding the random effects structure, a comparison was conducted between a model with random intercepts for participants and one that also incorporated random slopes for modality. Likelihood ratio tests indicated that the random-slope model provided a significantly better fit (χ^2^(2) = 224.20, *p* < 0.001), with lower AIC (4307.93 vs. 4528.13) and BIC (4374.72 vs. 4582.78). The final model included random slopes. To decompose significant interaction effects, simple effects analyses were conducted using the *emmeans* package, with Tukey’s HSD correction for multiple comparisons. Model convergence was verified through gradient inspection (maximum gradient < 0.01).

Model formula: log(RT*ijk*) = *β*0 + *β*1 ⋅ Group*i* + *β*2 ⋅modality*j* + *β*3 ⋅ (Group × modality)*ij* + *β*4 ⋅ IQ*i* + *β*5 ⋅ age*i* + *β*6 ⋅ PPVT*i* + *u*0*i* + *u*1*i* ⋅ modality*j* + *εijk*

In this formulation, *u*0*i* and *u*1*i* represent the random intercept for participants and the random slope for modality, respectively, with *εijk* denoting the residual error.

## 5. Results

### 5.1. Descriptive Statistics

#### 5.1.1. The Descriptive Statistics of Accuracy and RTs

Descriptive statistics for accuracy and RTs on the visual and auditory SL tasks are presented in Table 2.

#### 5.1.2. Model Predictions for Accuracy and RTs

**Figure 3:**
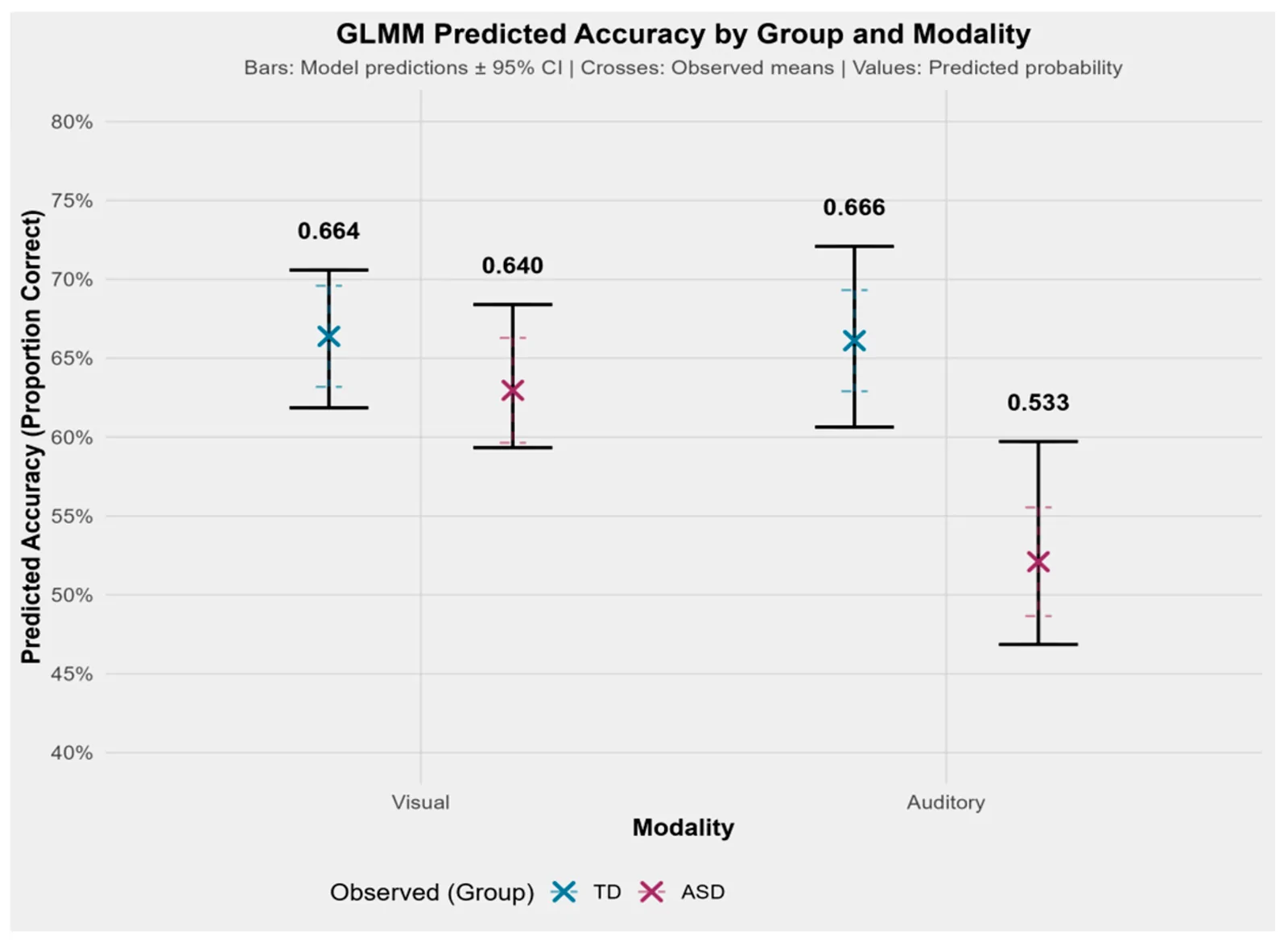
Predicted accuracy (with 95% CI) for the children with ASD and TD children on the visual and auditory statistical learning tasks.

Figure 3 displays the predicted accuracy and 95% CIs from the GLMMs. TD children showed relatively high accuracy in both modalities. In the visual task, children with ASD performed similarly to the TD children. However, in the auditory task, the predicted accuracy of children with ASD dropped significantly, falling below both TD children and their own visual-task performance.

**Figure 4:**
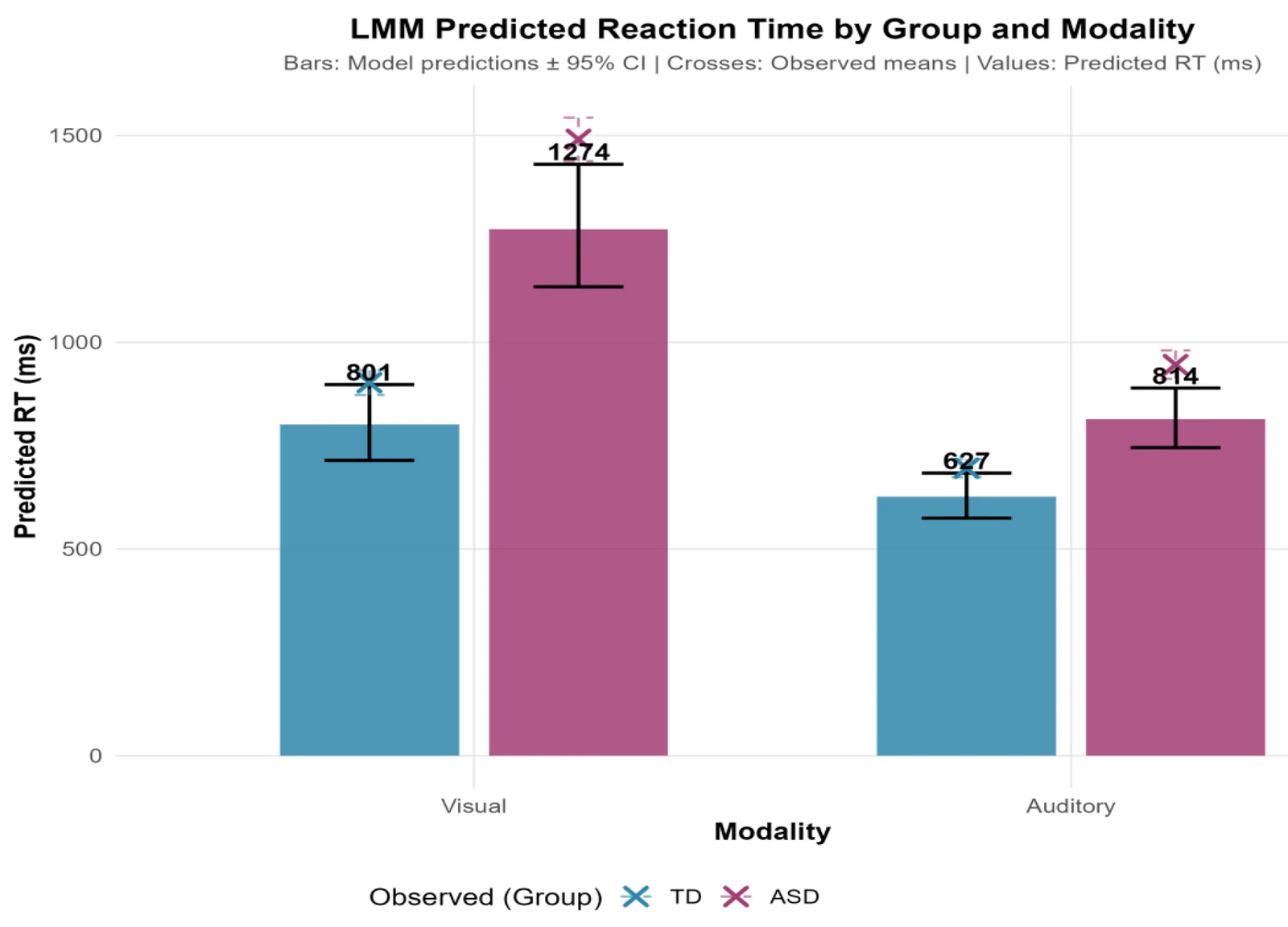
Predicted reaction time (with 95% CI) for the children with ASD and TD children on the visual and auditory statistical learning tasks.

Figure 4 presents the predicted RTs (with 95% CIs) from the LMM. Children with ASD exhibited slower predicted RTs compared to the TD children in both visual and auditory tasks. Additionally, both groups responded faster on the auditory task than on the visual task.

#### 5.1.3. Chance-Level Analyses

Chance-level analyses were conducted using a GLMM to determine whether predicted accuracy in each group significantly exceeded chance level (0.5) for each modality. Predicted probabilities and 95% CIs were extracted using the *emmeans* package, and Cohen’s *h* effect sizes were computed. Results showed the predicted accuracy of TD children was significantly above chance in both the visual (M = 0.664, 95% CI [0.620, 0.707], *p* < 0.001, Cohen’s *h* = 0.333) and auditory tasks (M = 0.666, 95% CI [0.609, 0.723], *p* < 0.001, Cohen’s *h* = 0.338). Similarly, children with ASD showed predicted accuracy significantly exceeding chance in the visual task (M = 0.640, 95% CI [0.594, 0.685], *p* < 0.001, Cohen’s *h* = 0.284). However, in the auditory task, their predicted accuracy did not differ significantly from chance (M = 0.533, 95% CI [0.469, 0.598], *p* = 0.156, Cohen’s *h* = 0.067).

#### 5.1.4. Individual-Level Analyses

**Figure 5:**
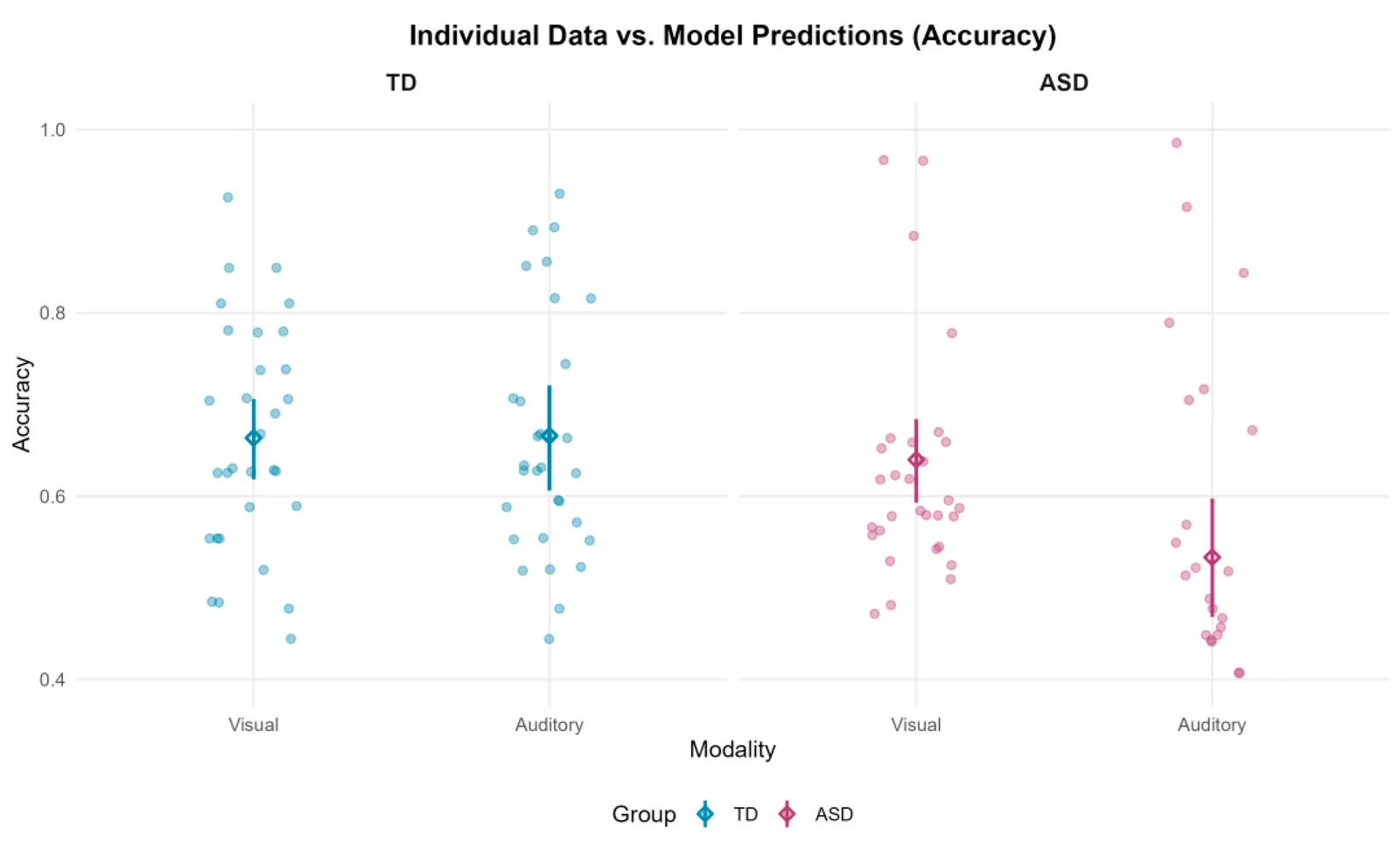
Observed versus predicted accuracy for individual participants across modalities.

To investigate the distribution of individual accuracy in the visual and auditory tasks, scatter plots were generated. These plots compare each participant’s observed accuracy with the model-predicted marginal means (Figure 5). Each point represents the average accuracy of a participant, while diamonds and error bars denote the model-predicted marginal means and 95% CIs, respectively. The figure demonstrates that, among TD children, the majority of participants’ accuracy in both modalities was concentrated above 0.5. Similarly, most children with ASD achieved accuracy exceeding 0.5 in the visual task. However, in the auditory task, a substantial proportion of ASD participants exhibited accuracy near or below 0.5, with a more dispersed distribution, indicating lower and more variable performance.

Further analysis examined the proportion of participants performing above chance. Among TD children, 87.1% (27/31) exceeded chance in the visual task, and 93.5% (29/31) did so in the auditory task. In the children with ASD, 93.3% (28/30) surpassed chance in the visual task, whereas only 40.0% (12/30) did so in the auditory task. Thus, the proportion of children with ASD exceeding chance was significantly lower in the auditory task than in the visual condition.

#### 5.1.5. Reliability Analysis of the Tasks

**Figure 6:**
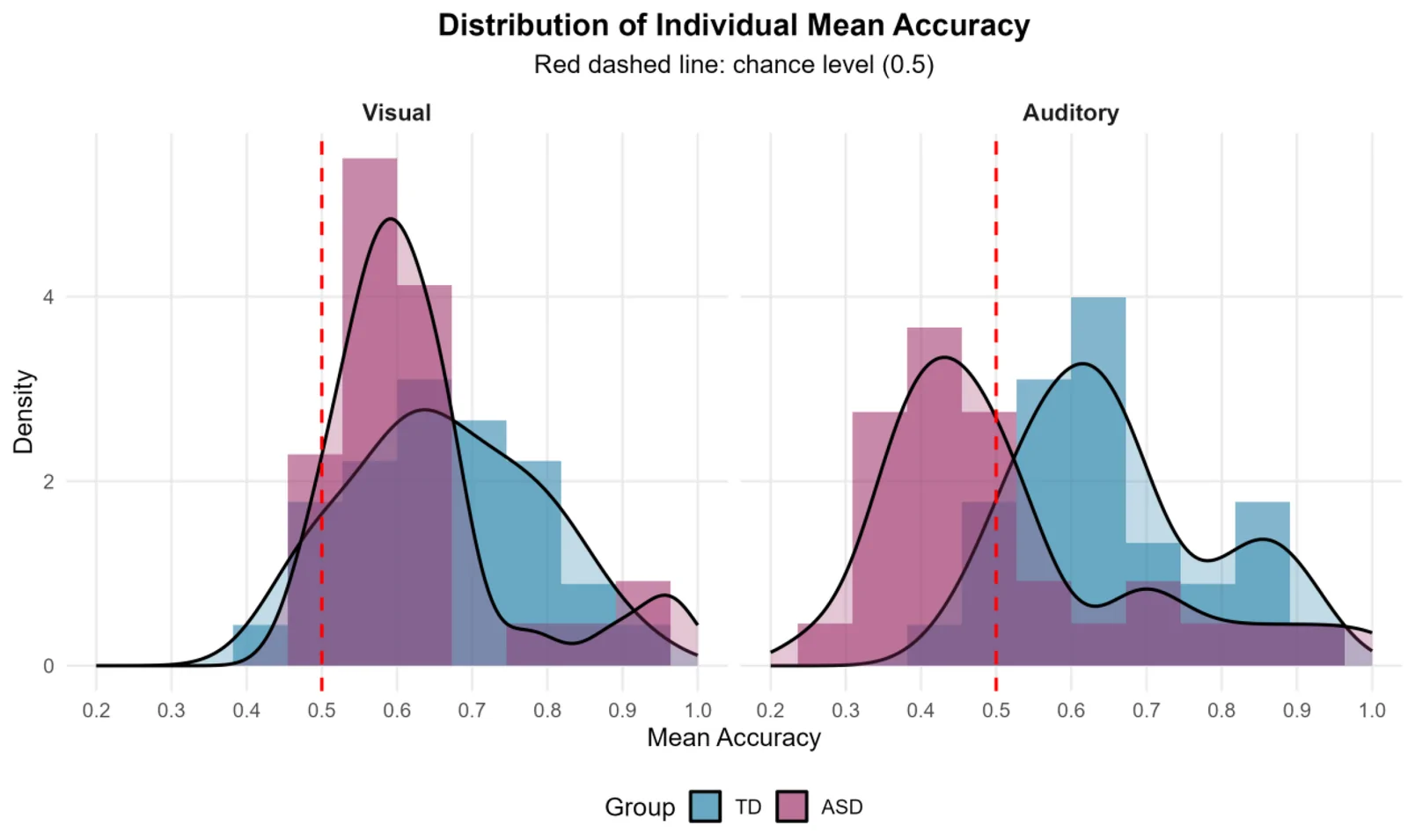
Mean accuracy distributions by group and modality.

The task comprised only 27 trials. To evaluate the psychometric properties of the task, analyses of split-half reliability, item difficulty, and performance distribution were conducted. Split-half reliability coefficients, corrected using the Spearman–Brown formula, were 0.72, 0.76, 0.65, and 0.50 across the four conditions (TD-visual, TD-auditory, ASD-visual, ASD-auditory). Item difficulty was assessed at both the target triplet level and the triplet combination level. At the target level, the accuracy for the three target triplets across both tasks ranged from 0.500 to 0.629, with no evidence of ceiling or floor effects. At the triplet combination level, nine target-interference triplet combinations were analyzed for each task. The accuracy for these combinations in the TD and ASD children ranged from 0.591 to 0.731 and from 0.367 to 0.689, respectively, with no extreme values observed. Regarding performance distribution, as shown in Figure 6, only children with ASD in the auditory task exhibited a positively skewed distribution, with most participants’ accuracy below 0.5; in all other conditions, the distribution centers were above 0.5.

### 5.2. Results of Statistical Learning Accuracy for Both Groups of Children

A GLMM was employed to analyze the effects of group and modality on accuracy. The model included group, modality, and interaction as fixed effects, with age, IQ, and PPVT-R as covariates, and modality specified as a random slope. The model yielded an AIC of 4282.2, a BIC of 4343.2, and a log-likelihood (LL) of −2131.1. Likelihood ratio tests comparing the full model to a simpler model without the interaction term revealed a significant interaction effect, *χ*^2^(1) = 4.93, *p* = 0.026. Furthermore, the full model showed a lower AIC (4282.2 vs. 4285.1; ΔAIC = 2.9). Consequently, the full model was adopted for analyzing accuracy (Table 3).

Table 3 shows a significant Group × Modality interaction on accuracy (*p* = 0.023). Simple effects analyses revealed that TD children showed no significant difference in accuracy between the two tasks (*OR* = 0.989, 95% CI [0.75, 1.30], *z* = −0.077, *p* = 0.938). However, children with ASD performed significantly better on the visual than on the auditory task (*OR* = 1.554, 95% CI [1.18, 2.05], *z* = 3.123, *p* = 0.002). In the auditory task, TD children outperformed children with ASD (*OR* = 1.74, 95% CI [1.21, 2.53], *z* = 2.949, *p* = 0.003). In the visual task, however, the two groups did not differ significantly (*OR* = 1.11, 95% CI [0.82, 1.47], *z* = 0.726, *p* = 0.468).

### 5.3. Results Analysis of SL Reaction Times for Both Groups of Children

An LMM was employed to analyze the effects of group and modality on RTs, with age, IQ, and PPVT-R as covariates, and modality as a random slope. The full model yielded an AIC of 4528.1, a BIC of 4582.8, and an LL of −2255.1. Likelihood ratio tests comparing the full model with a simpler model without the interaction term revealed a significant interaction effect, *χ*^2^(1) = 34.83, *p* < 0.001. Additionally, the full model showed a lower AIC (4528.1 vs. 4561.0; ΔAIC = 32.9). Consequently, RTs were analyzed with the full model (Table 4).

Table 4 shows a significant Group × Modality interaction on RTs (*p* = 0.020). Simple effects analyses revealed that TD children responded faster on the auditory than on the visual task (*β* = −0.244, *SE* = 0.059, *p* < 0.001). Similarly, children with ASD also responded faster on the auditory than on the visual task (*β* = −0.448, *SE* = 0.061, *p* < 0.001). Between groups, children with ASD were slower than TD children on both the auditory (*β* = 0.261, *SE* = 0.064, *p* < 0.001) and visual tasks (*β* = 0.464, *SE* = 0.084, *p* < 0.001).

### 5.4. Robustness Analysis

#### 5.4.1. Random Effects of Stimulus Materials

To assess whether variability at the triplet level influenced the results, a GLMM including a triplet as a random intercept was tested. The likelihood ratio test comparing this model (AIC = 4282.27, BIC = 4349.37, LL = −2130.14) to a model without the triplet random effect (AIC = 4282.17, BIC = 4343.17, LL = −2131.09) was not significant, χ^2^(1) = 1.90, *p* = 0.168. Accordingly, the triplet was not retained as a random intercept in the final model.

#### 5.4.2. Testing for Task Order Effects

To assess whether task order introduced systematic bias, an LMM was fitted with group, modality, and interactions as fixed effects, with task order included as a covariate. The analysis revealed no significant main effect of task order (*F*(1, 57.04) = 0.007, *p* = 0.933), nor any significant interactions involving task order. Specifically, task order × group (*F*(1, 57.00) = 0.084, *p* = 0.773), task order × modality (*F*(1, 57.00) = 0.636, *p* = 0.429), and task order × group × modality (*F*(1, 57.00) = 3.479, *p* = 0.067). These findings suggest that task order did not systematically bias the results of this study.

#### 5.4.3. Sensitivity Analysis of Aggregated Data

To confirm that the findings were not contingent on trial-level aggregation, a participant-level LMM was fitted with group as a between-subjects factor, modality as a within-subjects factor, and random intercepts for participants. The group-by-modality interaction was significant (*F*(1, 59) = 5.74, *p* = 0.020), consistent with the results obtained from the trial-level GLMM (*p* = 0.023). These results support the robustness of the main findings.

## 6. Discussion

The present study demonstrated that children with ASD exhibited deficits in ASL within a segmental paradigm. Specifically, their group-level performance did not significantly exceed chance levels, with only 40% of children with ASD achieving accuracy above the chance threshold. The GLMM analysis further revealed that their auditory learning performance was significantly inferior to that of TD children. Conversely, their VSL showed no significant difference from the TD children. RT analyses indicated that children with ASD displayed reduced processing efficiency in both modalities. However, their accuracy did not improve despite extended RTs of ASL.

### 6.1. Analysis of the Causes of Impaired ASL in Children with ASD

Children with ASD showed significantly lower accuracy than their TD children on the ASL task, despite having approximately 14% longer RTs (803 ms vs. 705 ms). These findings suggest reduced processing efficiency in the children with ASD, potentially reflecting elevated cognitive load or limited attentional resources during SL.

First, children with ASD often exhibit processing difficulties during the perceptual encoding stage, particularly in recognizing and extracting individual speech units from auditory streams. During early development, they often exhibit reduced orienting responses to speech sounds (e.g., human voices), while their processing of non-speech sounds remains relatively intact. This dissociation suggests that their difficulties may be domain-specific, primarily affecting the processing of auditory linguistic input (Dawson et al., 2004; O’Connor, 2012). Neuroimaging and electrophysiological studies have further demonstrated impaired sensitivity to changes in sound intervals at both brainstem and cortical levels. Additionally, these studies have revealed weakened auditory neural coding abilities (Sanglakh Ghoochan Atigh et al., 2024; Jansson-Verkasalo et al., 2003; Russo et al., 2009).

Second, limitations in auditory working memory have been proposed to impair the storage and retrieval of auditory information. Mayer and Heaton (2014) employed a word-for-word recall task, in which participants repeated sentences verbatim after hearing them. Their results indicated that, as speech rate increased, recall accuracy declined more rapidly in individuals with autism compared to TD children, which may be interpreted as a potential weakness in auditory working memory. Additionally, children with ASD exhibited difficulty identifying target sounds amid distracting noise during a triplet testing task. This finding may reflect challenges related to sound segregation and selective attention, although further research is needed to clarify the underlying mechanisms (Li et al., 2025).

Lastly, impaired auditory filtering may influence difficulties in ASL. When processing a high volume of repetitive stimuli, children with ASD often struggle to determine whether a given stimulus has been previously encountered, which may place excessive demands on cognitive resources for each novel item (Font-Alaminos et al., 2020). This increased cognitive demand could potentially contribute to sensory overload, which in turn may compromise their ability to automatically extract statistical regularities from auditory input (Poulsen et al., 2024). Collectively, these findings are consistent with the hypothesis that challenges related to auditory filtering and sensory overload may play a role in exacerbating social avoidance and communication difficulties, suggesting a possible cascading effect from fundamental perceptual abnormalities to higher-order cognitive and social impairments, although this cascading account awaits direct verification through longitudinal designs.

### 6.2. The Potential Impact of Impaired ASL on Language Abilities in Children with ASD

SL underpins various language abilities, including word segmentation, phonology, and semantic acquisition, with ASL being particularly relevant to language development (Parks et al., 2020; Liu et al., 2021). ASL depends on sensitivity to temporal intervals and boundary cues within sound sequences, with gap detection, defined as the ability to perceive the shortest silent interval in continuous sound, serving as a key index of this fundamental perceptual skill. In children with ASD, gap detection thresholds have been found to significantly correlate with receptive, expressive, and interactive language abilities, indicating a direct link between basic auditory temporal processing and higher-order language functions (Foss-Feig et al., 2017). It is proposed that ASL, particularly the extraction of regularities and the application of predictive coding, constitutes a core mechanism underlying language acquisition in ASD. This mechanism may impact word segmentation and syntactic processing, thereby influencing social communication and interactive discourse.

### 6.3. Analysis of the Factors Contributing to the Relative Strength of VSL in Children with ASD

Children with ASD demonstrated comparable accuracy to TD children on the VSL task, consistent with findings by Barnes et al. (2008) and Brown et al. (2010). However, their RTs were approximately 64% longer than those of TD children in the visual task (1614 ms vs. 982 ms), which may reflect generally slower processing speed rather than impaired learning per se. Nonetheless, this slower performance may be related to enhanced local visual processing. Mottron et al. (2006) observed that individuals with ASD tend to devote more attention to local features, such as the shape, position, and adjacency of objects, during visual processing. While this attentional bias might come at a cost in processing speed, it may also facilitate more precise segmentation of visual patterns from continuous input streams. Importantly, the triplet task employed in this study primarily involves perceiving transition probabilities and does not require integrating global visual information; thus, it may align well with the local processing style frequently observed in ASD. Neuroimaging studies have demonstrated increased activation of the occipital cortex during local visual processing in individuals with ASD (Huang et al., 2025), which may reflect neural mechanisms supporting this local processing advantage.

## 7. Conclusions

(1) Children with ASD exhibited selective difficulties in ASL, with their accuracy not significantly exceeding chance levels. This finding was consistent at the individual level.
(2) The performance of children with ASD in VSL tasks was relatively preserved, showing accuracy significantly above chance and no significant difference compared to the TD children.
(3) Children with ASD demonstrated generally slower RT across all tasks compared to the TD children.
(4) A significant group-by-modality interaction was found for both accuracy and RTs, indicating that these effects systematically varied depending on the task modality.

## 8. Limitations and Future Directions

First, the sample consisted of children with mild-to-moderate ASD (IQ > 70, CARS scores 30–36.5, and largely intact language abilities). The findings are primarily generalizable to high-functioning children with ASD and may not extend to those with severe cognitive impairments, limited or absent language, or significant comorbidities. Future research should include a broader ASD spectrum to evaluate the generalizability of ASL difficulties.

Second, the present findings, including both the auditory deficit and the visual preservation, were obtained using a segmented statistical learning paradigm with explicit blank intervals between successive triplets. Whether these modality-specific patterns generalize to other paradigms, such as artificial grammar learning or serial reaction time tasks, remains to be determined.

Third, while trait-level attention deficits were controlled, state-level attention during the familiarization phase was not assessed. Future studies could incorporate random distractors during the familiarisation phase. This would allow them to monitor attentional engagement in real time through behavioural responses (e.g., accuracy), thereby controlling for moment-to-moment attentional fluctuations that might influence learning performance.

Fourth, each testing condition involved only 27 trials, limiting repetition. Although reliability, difficulty, and distributional properties were examined, the limited trial count may reduce the stability of reliability estimates. Future studies should increase trial numbers to improve measurement reliability.

Fifth, the visual and auditory tasks used different stimulus types, alien images versus meaningless syllables, confounding modality with stimulus content. This confound may have contributed to the observed difficulties in ASL. Future studies could use cross-modal matching stimuli or vary stimulus types within the same modality to validate these findings.

Finally, this study relied on behavioral measures to infer modality-specific learning patterns and could not elucidate their neural underpinnings. Future research integrating electrophysiological techniques (e.g., ERPs) or neuroimaging methods (e.g., fMRI) could clarify whether auditory-specific deficits in ASD stem from perceptual encoding anomalies or difficulties in pattern integration.

Building on these findings, future research could focus on two main directions. First, whether ASL can serve as a diagnostic tool for language assessment warrants further investigation. Given its close association with language acquisition, subsequent studies could examine its predictive value for language development outcomes. Second, the potential of visual cues to support ASL merits additional exploration. Specifically, future research could evaluate whether providing visual supports such as images, text, or color coding alongside auditory input reduces cognitive load during ASL. Additionally, it could assess the feasibility and efficacy of this approach in naturalistic settings.

## Figures and Tables

**Table 1:** Demographic characteristics of the two groups and the results of group-matching tests (M ± SD).

	TD Children (*n* = 31)	Children with ASD (*n* = 30)	*t*	*p*	Cohen’s *d*	95% CI
Age	7.77 ± 0.51	8.05 ± 1.07	−1.283	0.205	−0.328	[−0.834, 0.177]
CRT	105.16 ± 9.96	100.77 ± 12.56	1.517	0.136	0.388	[−0.118, 0.895]
PPVT-R	73.12 ± 24.58	66.27 ± 24.33	1.095	0.278	0.281	[−0.224, 0.785]

**Table 2:** Descriptive Statistics for Accuracy and RTs in Visual and Auditory SL Tasks (M ± SD).

Groups	Modality	*n*	Accuracy	Original Reaction Time	Log Reaction Time
TD	Visual	31	0.664 ± 0.124	982 ± 457	6.78 ± 0.48
	Auditory	31	0.661 ± 0.130	705 ± 326	6.45 ± 0.47
ASD	Visual	30	0.628 ± 0.123	1614 ± 807	7.25 ± 0.55
	Auditory	30	0.521 ± 0.180	803 ± 390	6.57 ± 0.50

**Table 3:** Results of the GLMM on Accuracy in Visual and Auditory SL for Two Groups.

Fixed Effects	*β*	*SE*	*z*	*p*	*OR*	95% CI (OR)
Intercept	0.679	0.100	6.792	<0.001	1.973	[1.621, 2.400]
Group (ASD vs. TD)	−0.105	0.144	−0.726	0.468	0.901	[0.679, 1.194]
Modality (Auditory vs. Visual)	−0.011	0.141	−0.077	0.938	1.011	[0.767, 1.333]
Group × Modality	−0.452	0.199	−2.272	0.023	0.636	[0.431, 0.940]
						
IQ	0.001	0.006	0.249	0.803	1.001	[0.990, 1.013]
Age	−0.028	0.080	−0.353	0.724	0.972	[0.832, 1.136]
PPVT-R	0.004	0.003	1.560	0.119	1.004	[0.999, 1.010]
**Random Effects**	Variance	***SD***				
Modality slope	0.266	0.516				

Note: OR = Odds Ratio; CI = Confidence Interval; Group reference = Typically Developing (TD); Modality reference = Visual; All covariates were mean-centered.

**Table 4:** Results of the LMM on RTs in Visual and Auditory SL for Two Groups of children.

Fixed Effects	*β*	*SE*	df	*t*	*p*	95% CI
Intercept	6.686	0.058	59.6	114.862	<0.001	[6.572, 6.800]
Group (ASD vs. TD)	0.464	0.084	60.9	5.528	<0.001	[0.300, 0.629]
Modality (Auditory vs. Visual)	−0.245	0.059	58.9	−4.120	<0.001	[−0.362, −0.129]
Group × Modality	−0.203	0.085	59.1	−2.390	0.020	[−0.369, −0.037]
						
IQ	−0.009	0.003	56.2	−3.415	0.001	[−0.014, −0.004]
Age	−0.046	0.036	56.0	−1.269	0.210	[−0.118, 0.025]
PPVT-R	0.003	0.001	56.0	2.149	0.036	[0.001, 0.005]
**Random Effects**	Variance	***SD***				
Subject	0.094	0.307				
Residual	0.205	0.453				

Note: All covariates were mean-centered; response times were log-transformed.

## Data Availability

The raw data supporting the conclusions of this article will be made available by the authors on request.
